# Supplementary material for: Structural basis of voltage-dependent gating in BK channels
Source: Nat Commun. 2025 Jul 1;16:5846. doi: 10.1038/s41467-025-60639-y (PMC12218146; doi:10.1038/s41467-025-60639-y)
Supplement: Supplementary file 1 — Supplementary Information [file 41467_2025_60639_MOESM1_ESM.pdf]

Supplementary Information

Supplementary Figure 1. Flow chart for the Cryo-EM data processing and structure determination for R1Q.

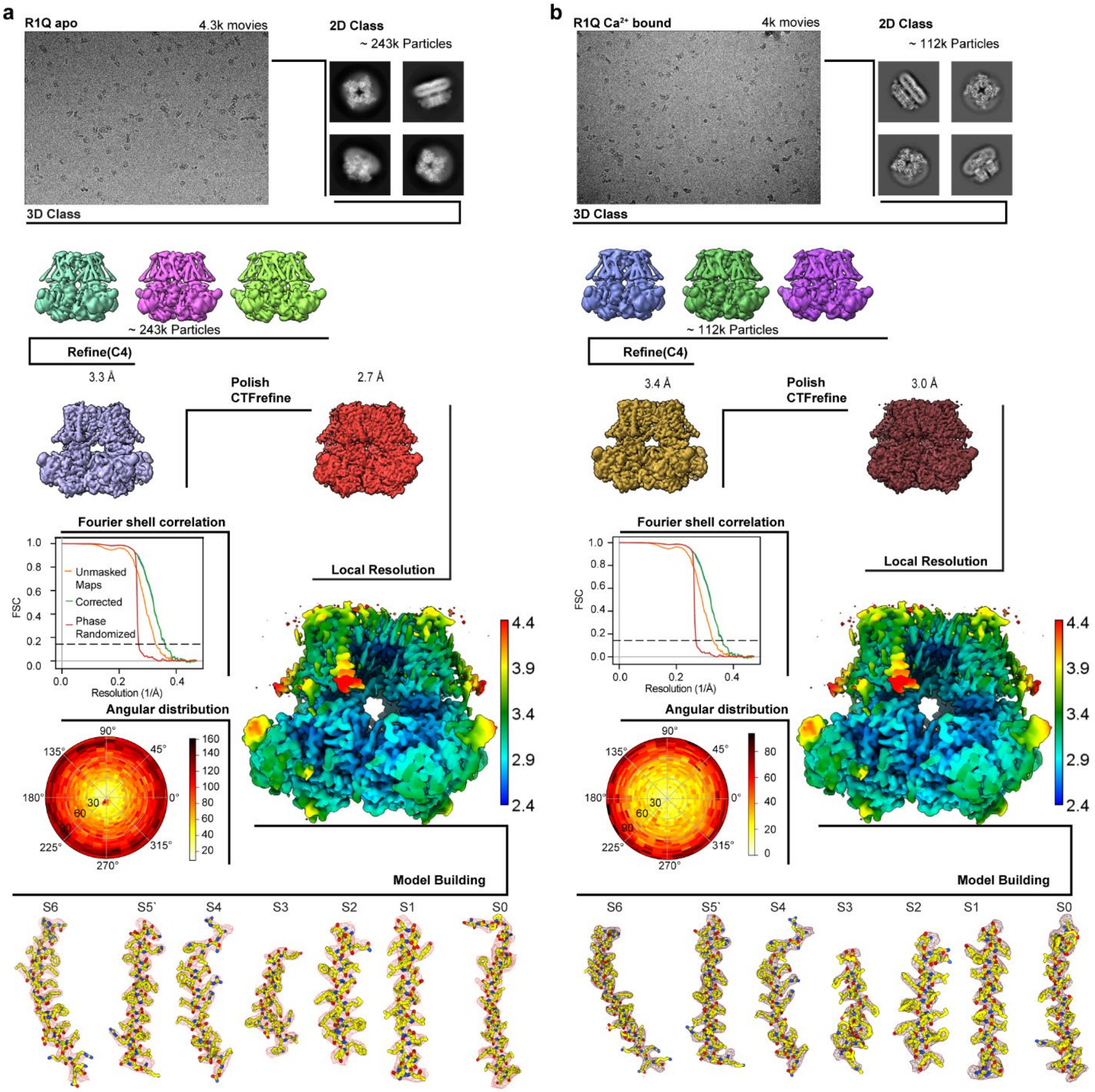

a-b. Fourier shell correlation (FSC) plot of the refined maps, with resolution determined using the FSC 0.143 criterion. **Angular distribution** plot for the refined full molecule is shown. **Local resolution** estimation is visualized using ResMap coloring on the unfiltered half map. **Model building** details are illustrated with representative segments of the sharpened cryo-EM density map overlaid with the fitted atomic model.

Supplementary Figure 2. Flow chart for the Cryo-EM data processing and structure determination for R2Q.

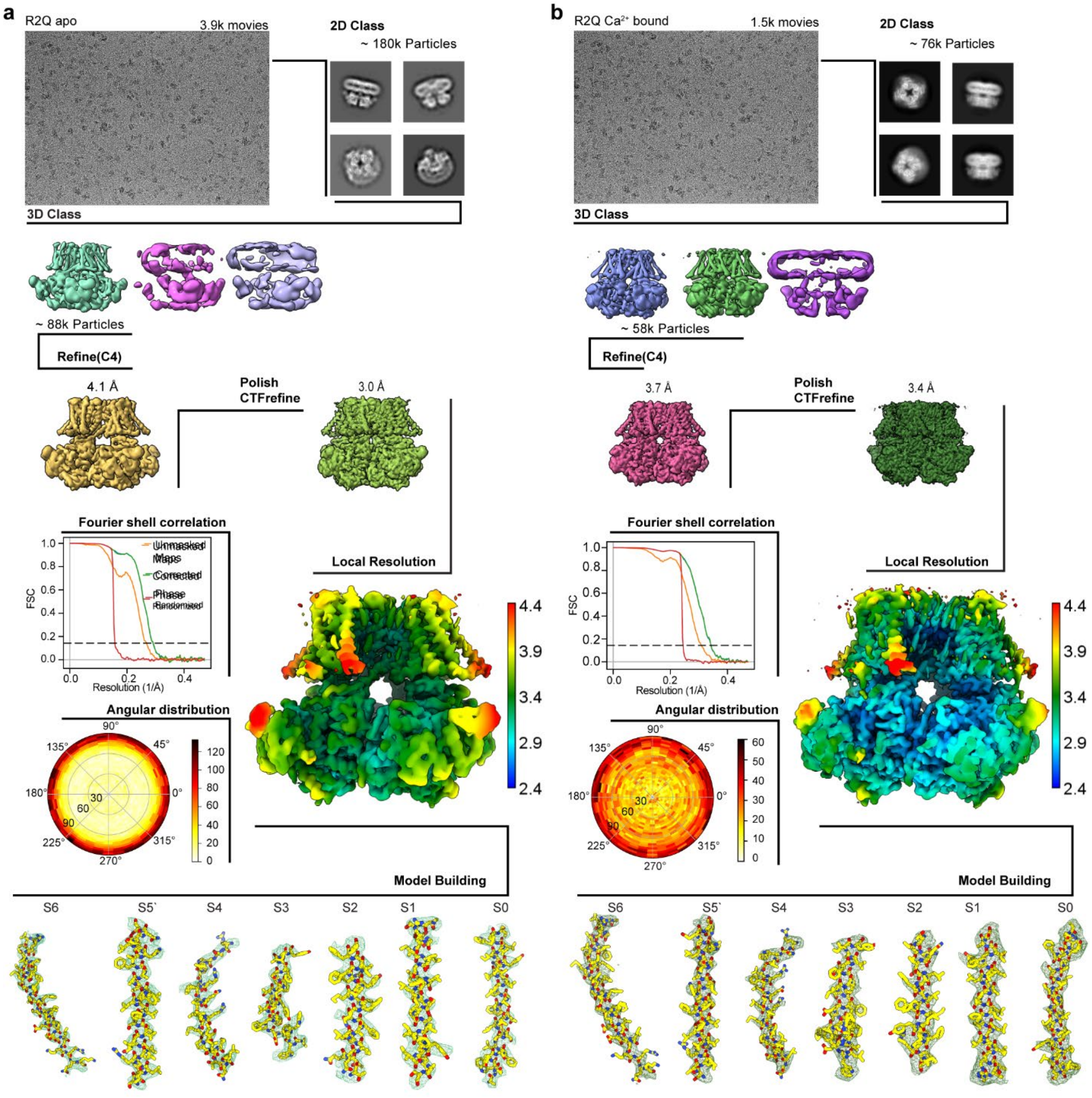

a-b. Fourier shell correlation (FSC) plot of the refined maps, with resolution determined using the FSC 0.143 criterion. **Angular distribution** plot for the refined full molecule is shown. **Local resolution** estimation is visualized using ResMap coloring on the unfiltered half map. **Model building** details are illustrated with representative segments of the sharpened cryo-EM density map overlaid with the fitted atomic model.

Supplementary Figure 3. Flow chart for the Cryo-EM data processing and structure determination for R3Q.

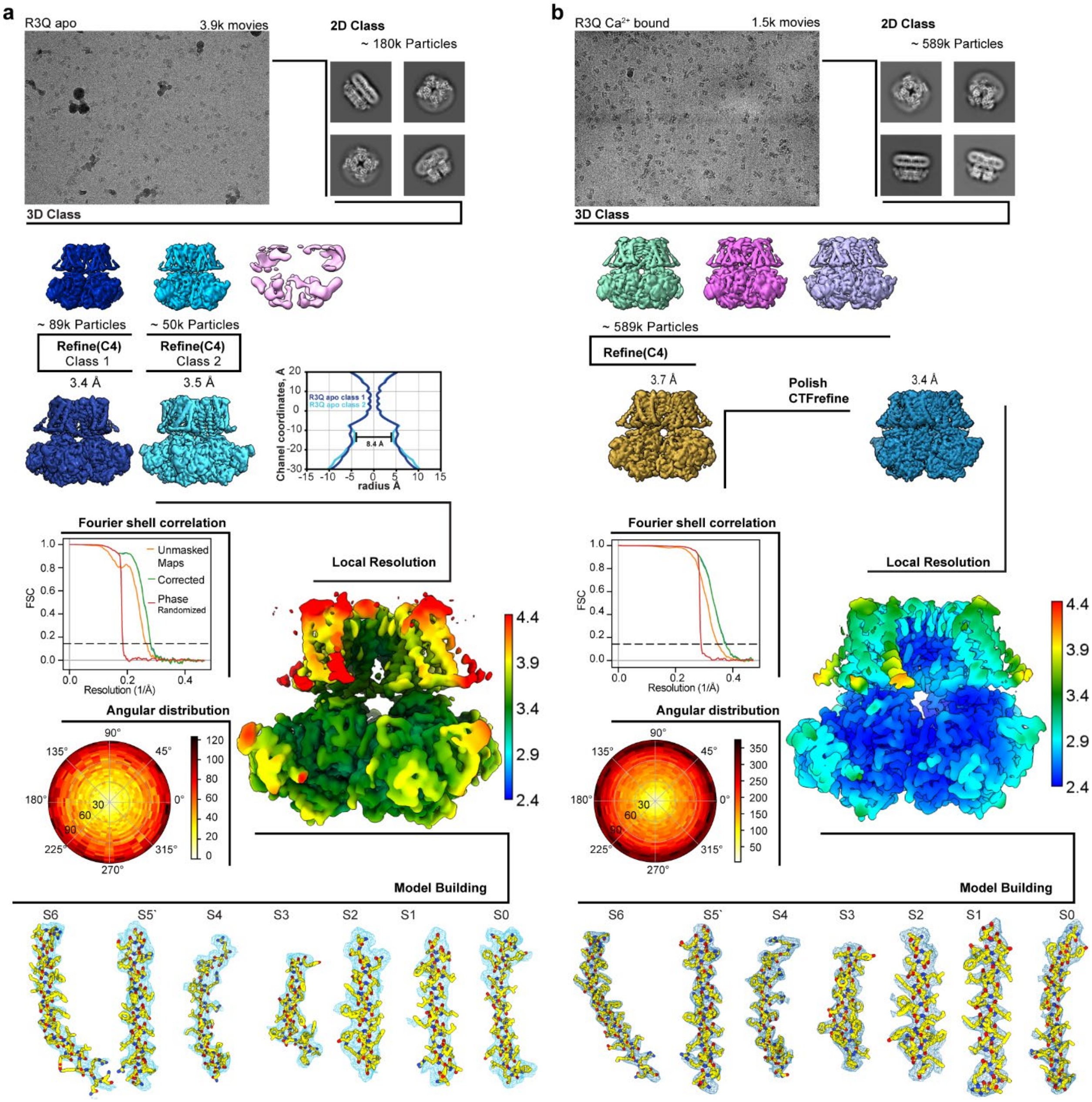

a-b. Fourier shell correlation (FSC) plot of the refined maps, with resolution determined using the FSC 0.143 criterion. **Angular distribution** plot for the refined full molecule is shown. **Local resolution** estimation is visualized using ResMap coloring on the unfiltered half map. **Model building** details are illustrated with representative segments of the sharpened cryo-EM density map overlaid with the fitted atomic model.

Supplementary Figure 4. Flow chart for the Cryo-EM data processing and structure determination for F304A.

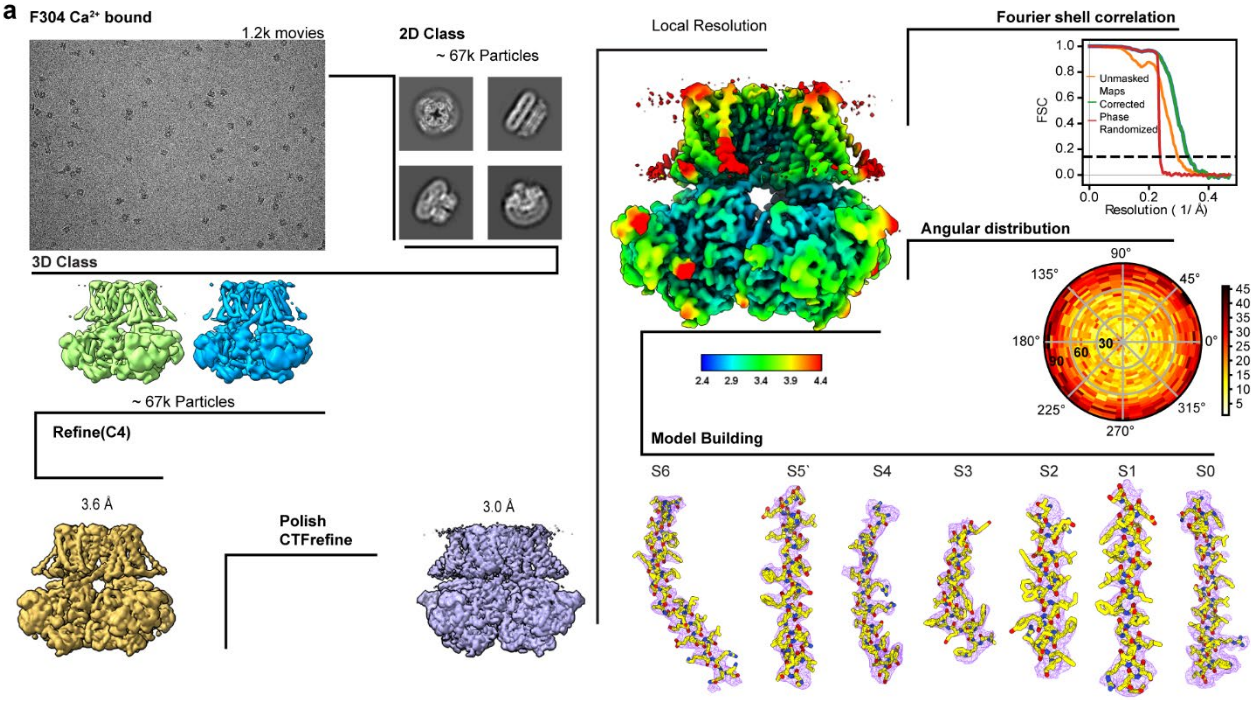

a. Fourier shell correlation (FSC) plot of the refined maps, with resolution determined using the FSC 0.143 criterion. **Angular distribution** plot for the refined full molecule is shown. **Local resolution** estimation is visualized using ResMap coloring on the unfiltered half map. **Model building** details are illustrated with representative segments of the sharpened cryo-EM density map overlaid with the fitted atomic model.

Supplementary Figure 5. Intramolecular interactions in the VSD of aBK.

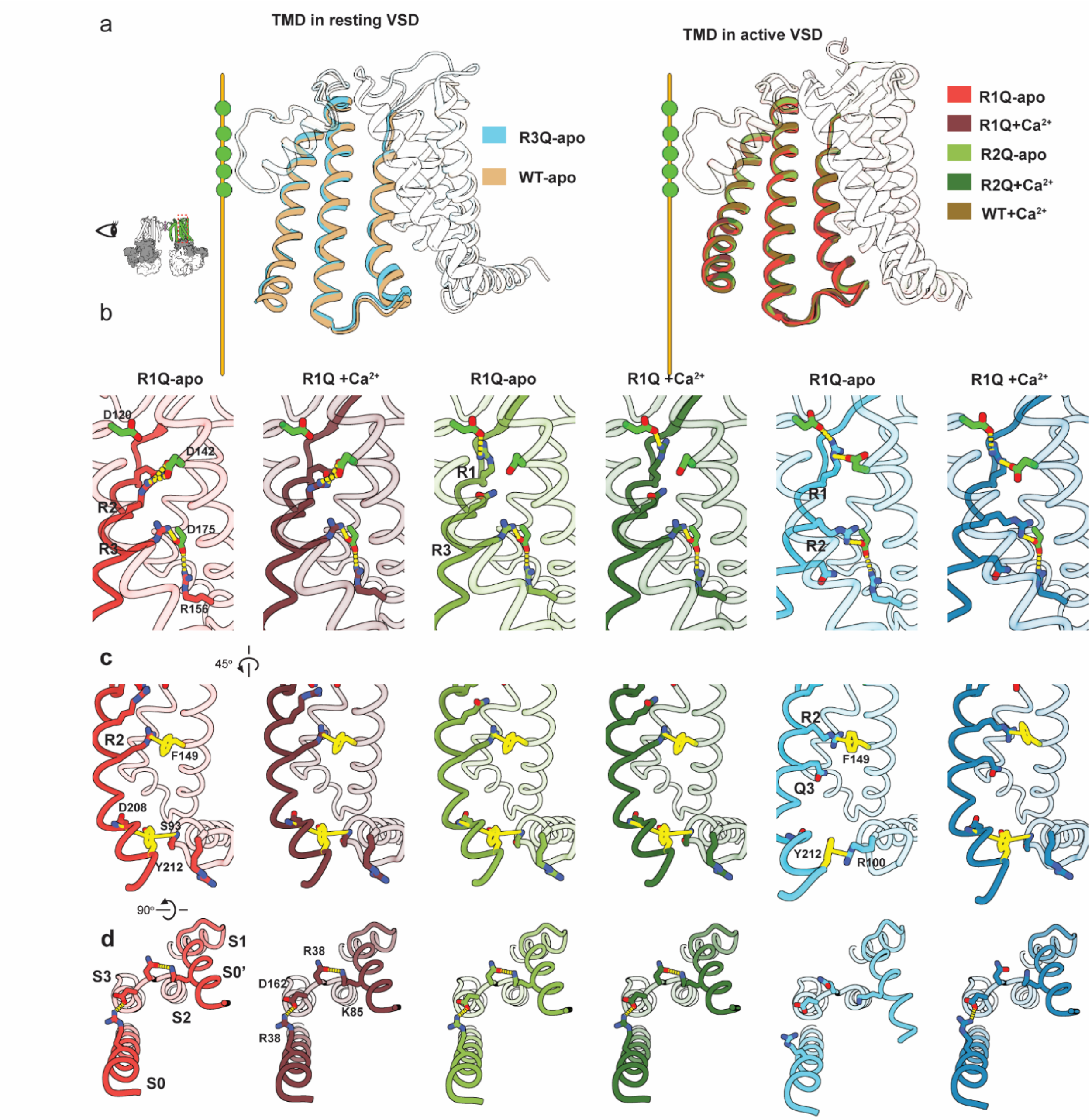

a, Schematic representation showing between the TMD of R3Q and WT apo, and open channel structures R1Q and R2Q with WT Ca^{2+}-bound. **b**, side view of the charged residues of the VSD and the electrostatic interactions. **c**, side view of the charged residues of the VSD and their state dependent cation-π (R2-F149,R3-F149 and R100-Y212) or amide-π (D208-Y212-S93) interactions. **d**, bottom view of VSD.

Supplementary Figure 6. Negative residues set an acidic environment in the VSD of BK.

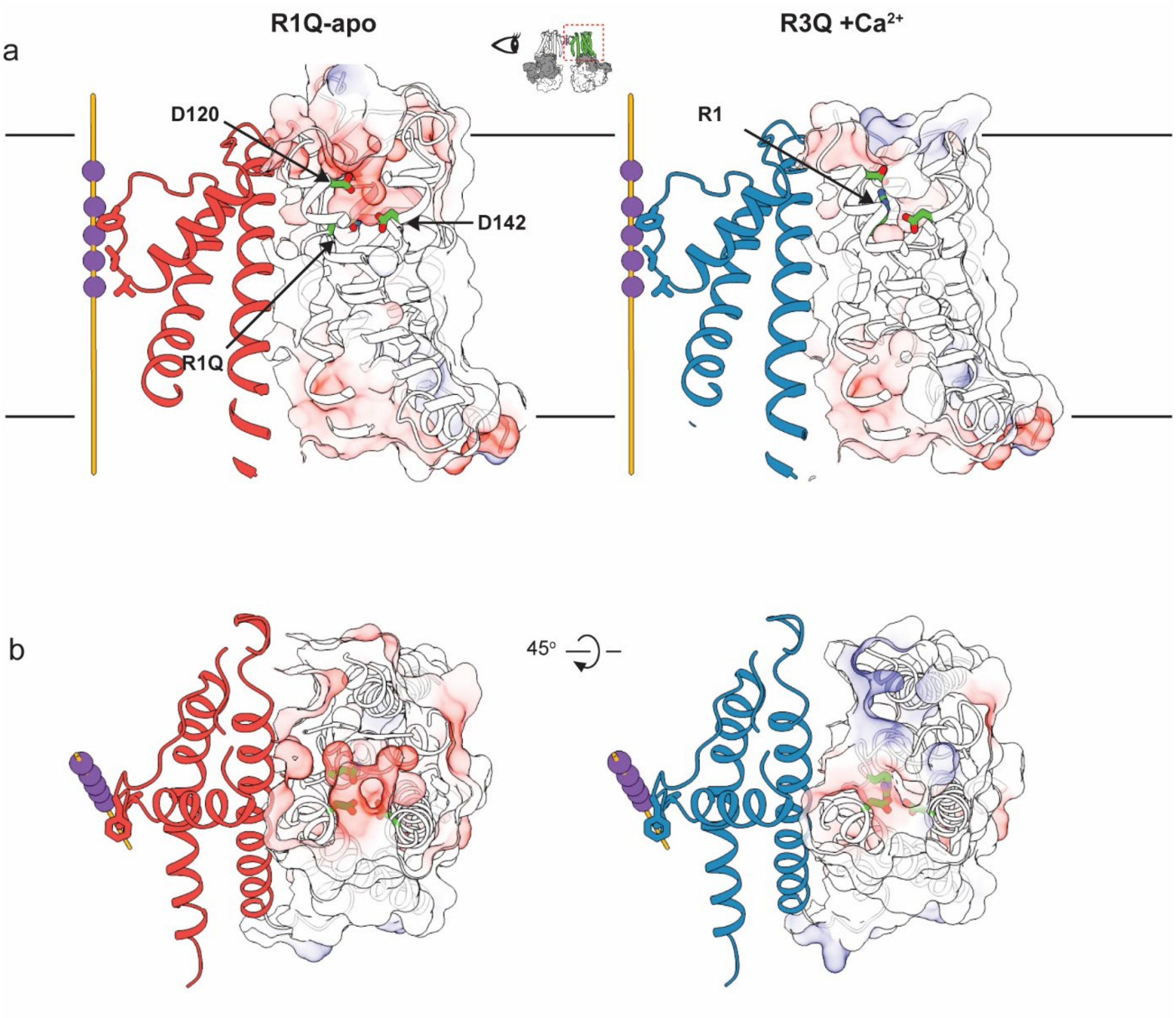

a and **b**, Electrostatic potential surfaces of the VSD (residues 1-218) of the R1Q-apo (*left*) and R3Q Ca^{+2}-bound (*right*) structures. Key residues R1 (Q196 *left* and R196 *right*), D120, and D142 are shown as sticks.

Supplementary Figure 7. New structural features in active VSD.

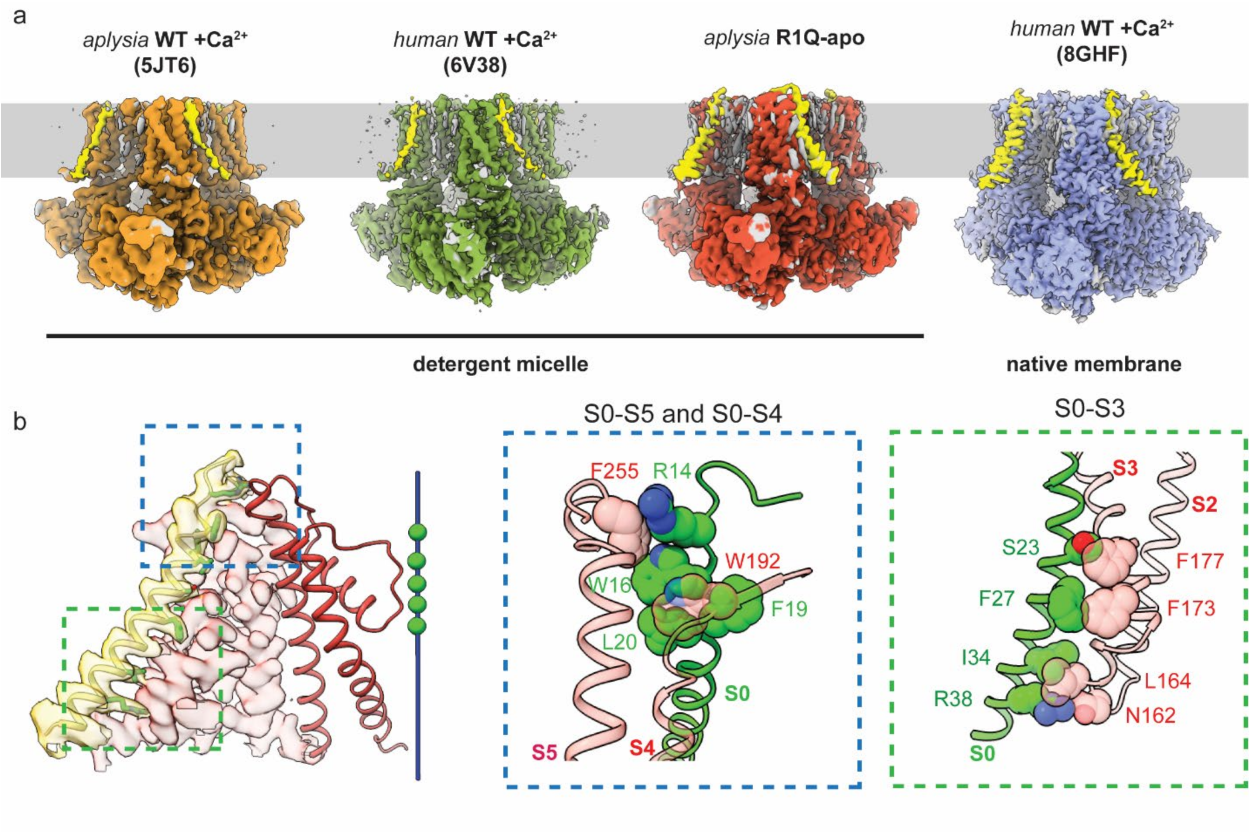

a Cryo-EM maps of BK structures with active VSD in detergent aBK-WT Ca+2-bound (orange PDB 5TJI), hBK-WT Ca+2-bound (green PDB 6V38), aBK-R1Q-apo (red), and native membranes hBK-WT Ca+2-bound (purple PDB 8GHF). Segment S0 is yellow. b, Composite cryo-EM map and structural model of R1Q-apo shown in ribbon representation; well-resolved residues in S0 are in close contact with the rest of the VSD. The extracellular side residues of S0, R14, W16, F19, and L20 (green spheres), are in steric contact with W196 in S4 and F255 in S5 (red spheres). In contrast, at the cytoplasmic end of S0, S23,F27, I34, and R38 (green sphere) interact with F177,F173, L164, and N162 (red) in S3.

Supplementary Figure 8. VSD induces conformational changes in the CTD .

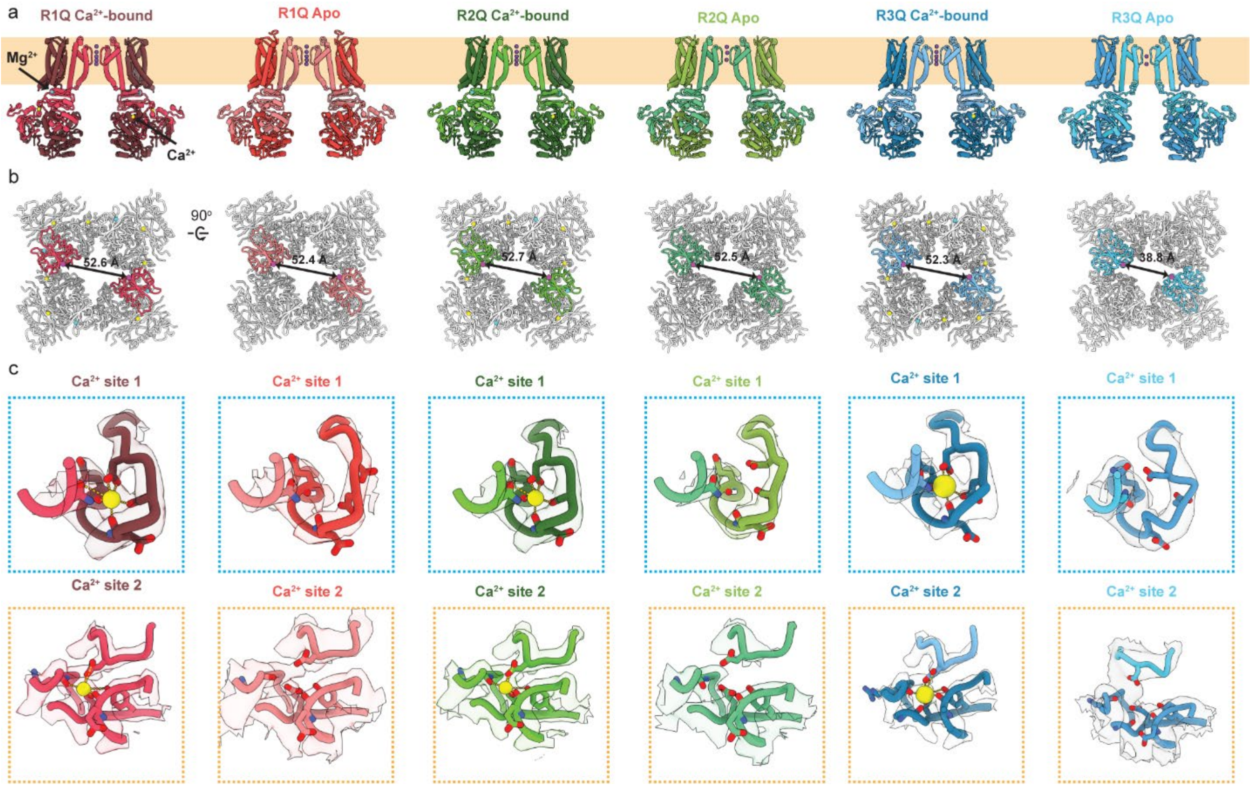

a, Side of the atomic models of R1Q-apo (red, *left*) and R1Q Ca^{+2}-bound (crimson, right); R2Q-apo(green light) and R2Q Ca^{+2}-bound (olive) (middle); R3Q-apo(light blue) and R3Q Ca^{+2}-bound (steel blue) (left). Calcium ions are colored yellow. b, top of the gating ring, for simplicity only two out of for RCK1 domain were colored. **c**, Composite view of the Cryo-EM maps and atomic model focused on the two calcium-binding sites at the CTD.

Supplementary Figure 9. Changes in hydrophobicity in aBK permeation pathway.

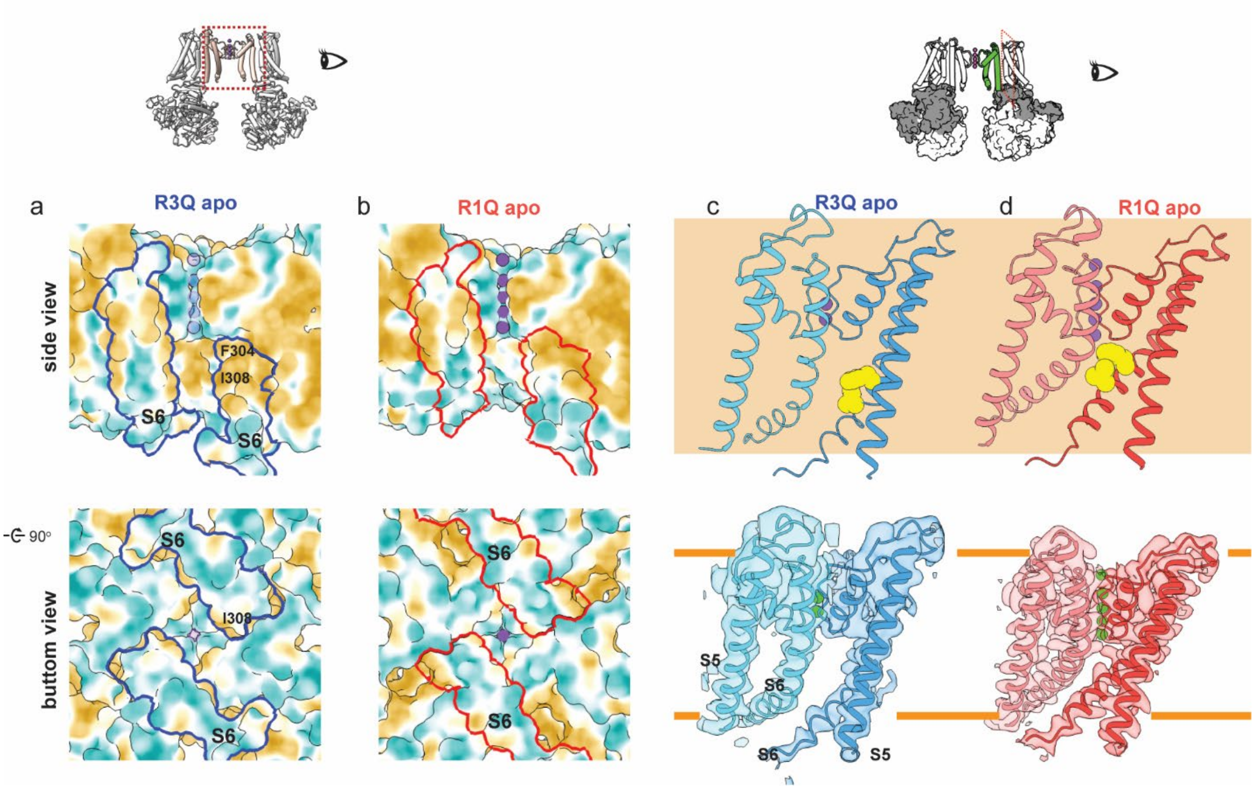

a and **b**, Side and top view of the ion permeation pathway of R3Q (left) and R1Q (right) in the apo condition solved in the presence of DDM, shown in surface representation where the surface is colored based on lipophilicity, from gold (lipophilic) to blue (hydrophilic). **c** and **d**, Peripheral side view of pore domain of two contiguous monomer, shown in cartoon representation including segments S4-S6, comparing the closed channel R3Q-apo (left light blue) with the open R1Q-apo (left red). The hydrophobic residues F304 and I308 are shown in the vdW representation.

Supplementary Figure 10. bbTBA as geometrical constrain for BK permeation pathway.

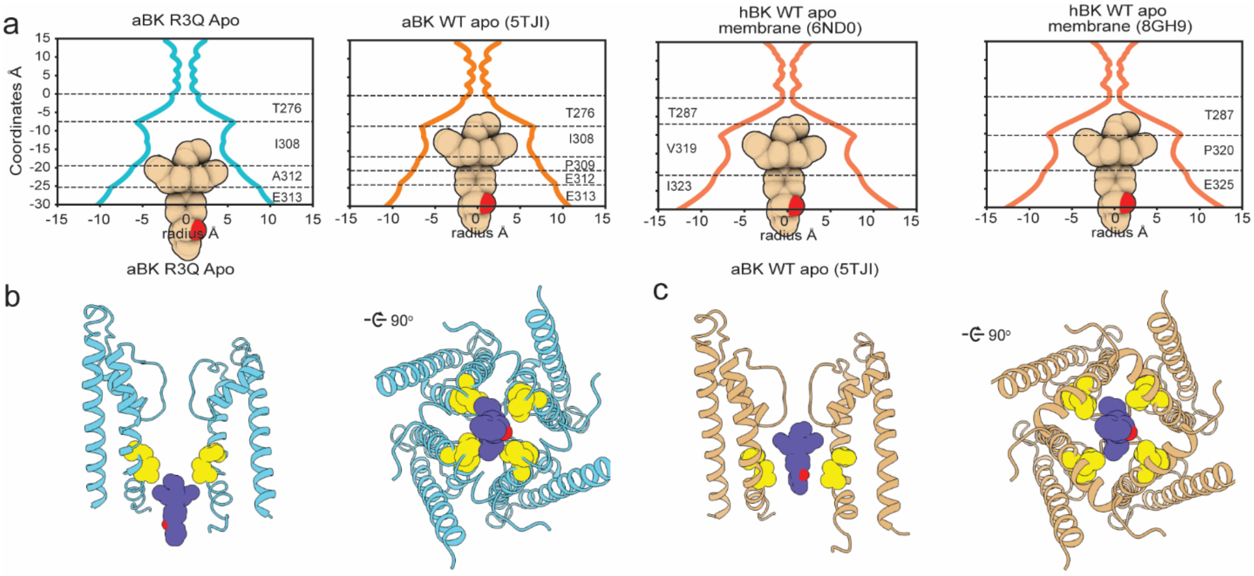

a, Comparation of the pore radius for aBK-WT apo (5TJI) solved in detergent, as well as hBK-apo (6ND0) and hBK apo (8GH9) in native membrane. **b**, Pore radius for metal free *aBK R3Q* structure. **c** and **d**, Side (left) and bottom(right) view of PGD including segments S4-S6 of aBK-WT apo (**c**, 5TJI) and R3Q-apo (**d**) overlay with the atomic model of bbTBA.

Supplementary Figure 11. R3Q-apo unfolds the N-terminus of αB/RCK1.

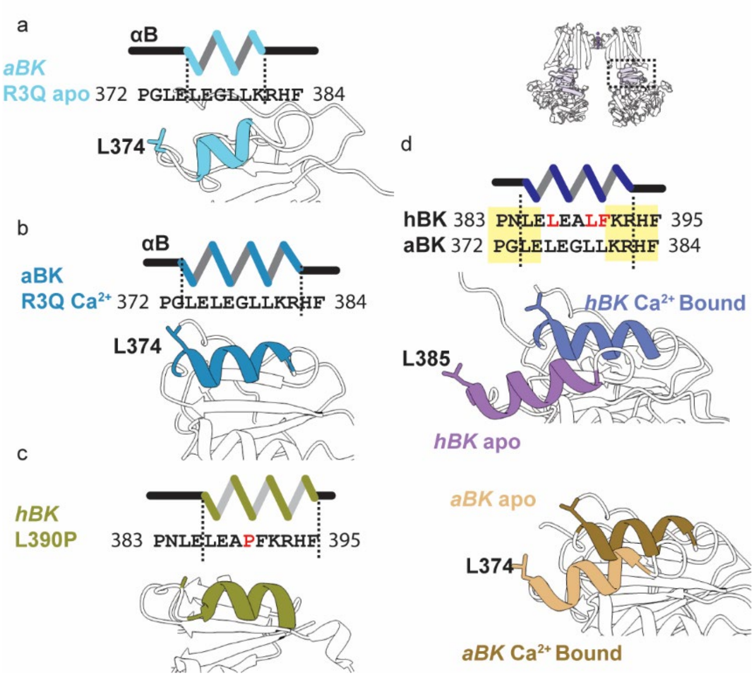

a-c, The structural comparison between the αB helix in the CTD of both aBK (R3Q-apo and Ca^{+2}-bound) and hBK –L380P (PDB 6V5A). **d**, the αB helix in the CTD of hBK under Ca^{+2}-bound conditions (PDB 6V38) and apo conditions (PDB 6V3G).

Supplementary Figure 12. RCK1 rearrangement influences the structure of the resting VSD.

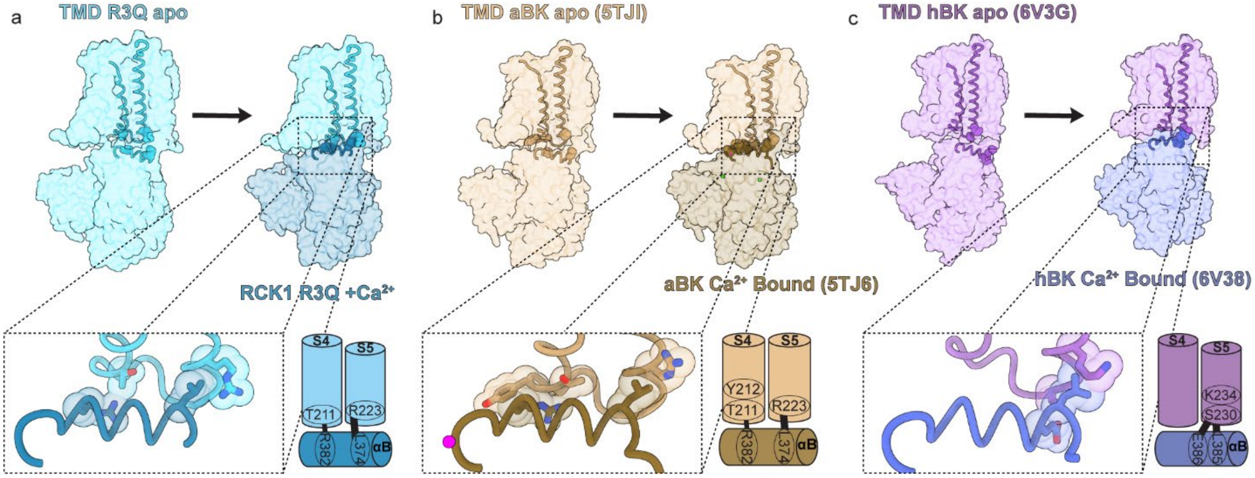

a, composite view of the TMD and the CTD of two contiguous monomers, of R3Q-apo (*top left*) followed the overlay of the CTD R3Q Ca^{+2}-bound (*top right*). The TMD's segment S4 and S5 shown in cartoon representation, as well as the αB helix at CTD. The TMD and CTD are shown as a transparent surface. Key residues are display vdW representation. (*bottom left*) zoom of the interaction surface between the metal bound-CTD and the resting VSD. The residues producing clashes between are display in stick representation overlay with their vdW representation. (*bottom right*) schematic representation of the interacting residues an their secondary structure.

b, comparation between TMD of WT-apo (5TJI) and the CTD of WT Ca^{+2}-bound (5TJ6).

c, comparation between TMD of hBK-apo (6V3G) and hBK Ca^{+2}-bound (6V38).

Supplementary Figure 13. VSD rearrangement influences the structure of the unbound gating ring.

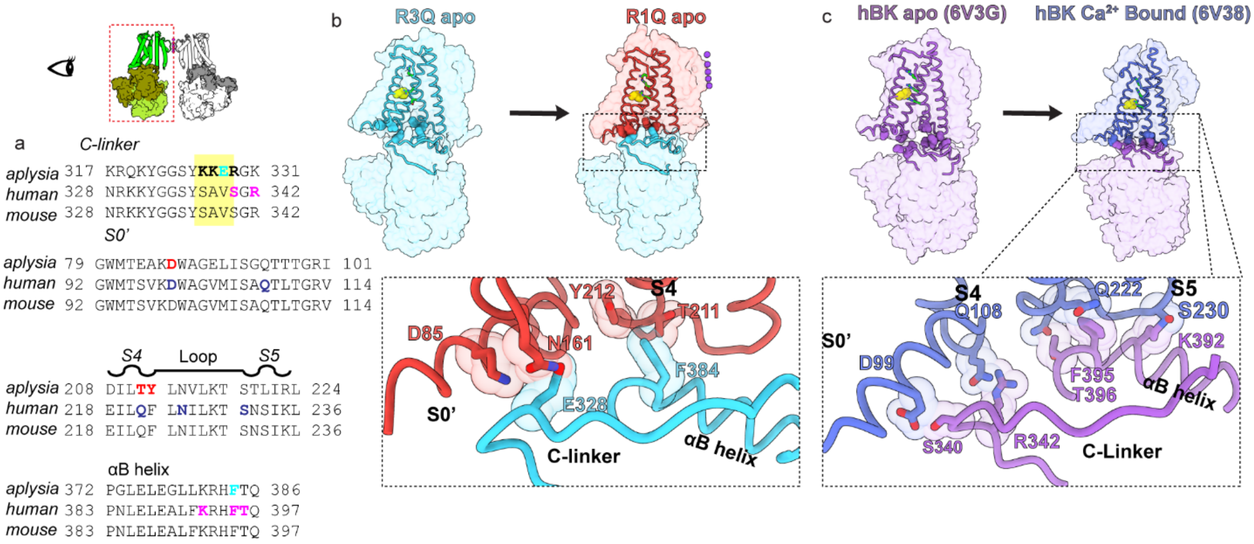

a, Sequence alignment of the secondary structures to be compared. **b** and **c**, structural comparation superimposing the structure of the active VSD of R1Q-apo with the CTD of R3Q apo (b), and the VSD of hBK Ca^{+2}-bound (6V38) with the CTD of hBK-apo (6V3G).

Supplementary Figure 14. Structural features of F304A Ca^{2+}-bound.

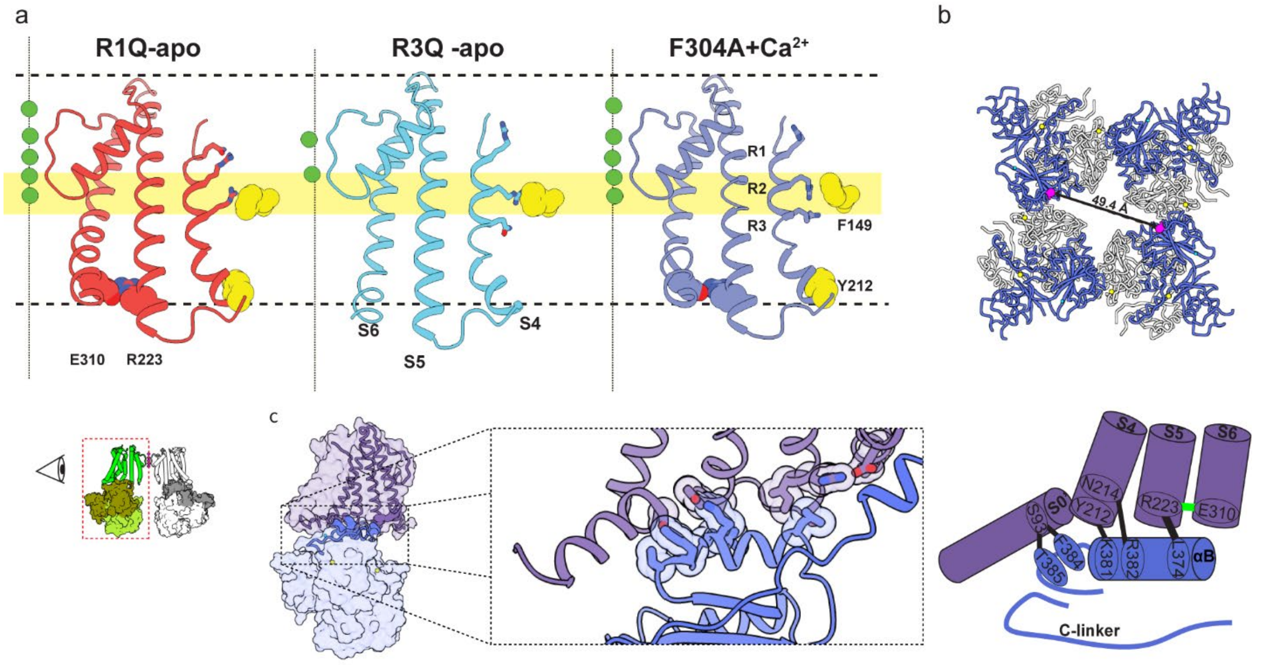

a, comparation of the position of S4 charged residues with respect to F149, for R1Q-apo, R3Q-apo, F304A Ca^{2+}-bound. b, top view of the gating ring of F304A Ca^{2+}-bound. c, composed view of two adjacent α-monomers. The TMD and RCK1 are represented in a cartoon and the structure of the whole monomer is overlay is represented as a transparent surface. (*middle*) zoom of the interaction surface between VSD-CTD, the key interacting residues are shown as stick representation superimposed with their transparent sphere representation. (bottom) scheme highlighting the key residues stabilizing the interaction between VSD-CTD.

Supplementary Figure 15. Configuration of S6 affects the intersubunit coupling in the internal cavity of BK channels.

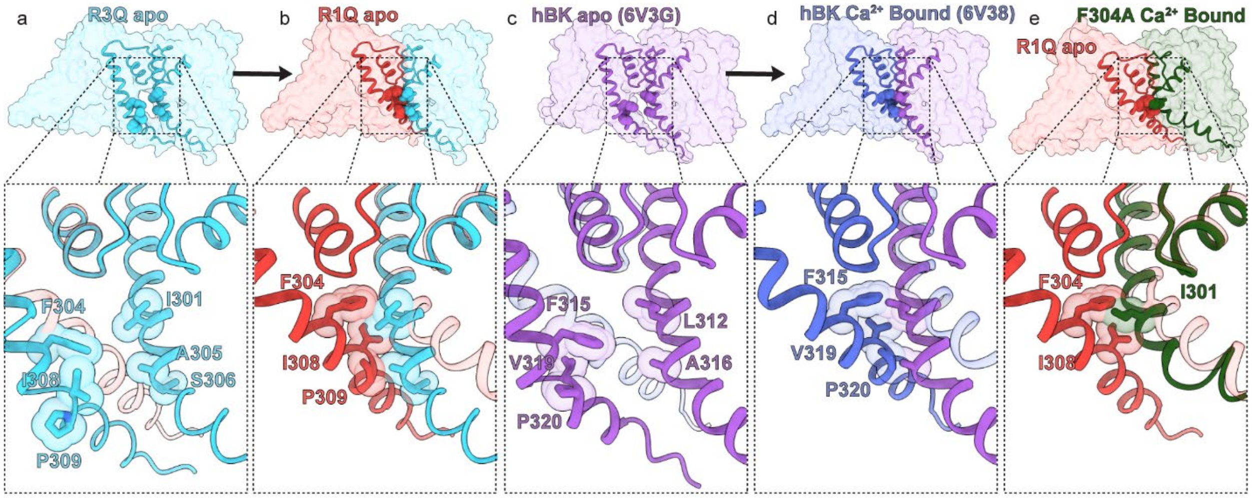

a-e, Composed view of two adjacent α-monomers. The S6 helix of each monomer are illustrated in a cartoon and the structure of the whole monomer is shown as a transparent surface. (*top*) zoom of the interaction surface between the S6 adjacent α-monomers, the key interacting residues are shown as stick representation superimposed with their transparent sphere representation.

Supplementary Table 1. Cryo-EM data collection, refinement and validation statistics

Name	R1Q		R2Q		R3Q		F304A
Mutation	R196Q		R199Q		R202Q		F304A
Condition	Apo	Ca^{2+}-bound	Apo	Ca^{2+}-bound	Apo	Ca^{2+}-bound	Ca^{2+}-bound
PDB code	9DIC	9DI8	9DJV	9DIT	9DKN	9DKF	9DKL
EMDB	EMD-46903	EMD-46901	EMD-46939	EMD-46918	EMD-46963	EMD-46956	EMD-46961
**Data Collection**							
Microscope	Titan Krios 300 kV						
Detector	K3 summit (Gatan)						
Magnification	81,000						
Defocus Range	0.5-2.2 (μm)						
Total dose	65 e^{-}/ $Å^2$						
Number of frames	50	50	50	50	50	50	50
Pixel size (Å/pixel)	0.5315	0.5315	0.5315	0.5315	0.536	0.5315	0.5315
Number of movies	4063	4002	5798	1791	3304	8230	1173
**Data processing**							
Number of particles refined	243K	111K	88K	57K	116K	589K	65K
Symmetry	C4	C4	C4	C4	C4	C4	C4
Resolution (Å)	2.7	2.9	3.4	2.9	3.6	2.6	3
sharpening B-factor ($Å^2$)	-36	-79	-114	-85	-146	-99	-83
FSC threshold	0.143	0.143	0.143	0.143	0.143	0.143	0.143
Initial model (PDB ID)	5TJ6	5TJ6	5TJ6	5TJ6	5TJI	5TJ6	5TJ6
model resolution (Å)	2.6	2.6	2.9	2.6	3.5	3	2.7
Model Composition							
non-hydrogen atoms	28433	28220	28207	28221	28376	27861	28418
Protein residues	3620	2596	3596	3596	3596	3560	3596
Ligands	5	17	3	17	0	17	10
B factors($Å^2$)							
Proteins	113.61	114.15	114.15	114.15	62.81	221.89	137.65
Ligands	61.05	91.85	65.68	101.57	n/a	198.19	121.29
R.M.S. deviations							
Bond lengths (Å)	0.012	0.012	0.011	0.011	0.011	0.011	0.014
Bond angles(°)	1.813	1.852	1.763	1.814	1.812	1.807	1.826
Validation							
Molprobity score	1.26	1.28	1.21	1.13	1.61	1.3	1.7
Clashscore	2.4	2.95	1.92	1.7	4.58	3.73	6.7
Poor rotamers (%)	0.75	0.93	0.66	0.6	0.82	0.41	0.78
Ramachandran plot							
Favored(%)	96.42	96.84	96.25	96.73	94.53	97.29	95.29
Allowed(%)	3.56	3.16	3.75	3.27	5.38	2.71	4.62
Disallowed(%)	0.03	0	0	0	0	0	0.08

Supplementary Table 2. Molecular dynamics simulation system setup

System	WT apo (PDB ID: 5tji)	R3Q apo class1 (PDB ID: 9DKN)
Simulation box dimensions	100×100×105 $Å^3$	100×100×105 $Å^3$
Total number of atoms	96,181	99,905
Total number of water molecules	19,345	20,495
Salt concentration	150 mM KCl	150 mM KCl
Lipid type	POPC	POPC
Lipid number: outer leaflet	112	114
Lipid number: inner leaflet	123	123

Supplementary Table 3. Reliability and reproducibility checklist for molecular dynamics simulations.

	Yes	No	Response
**1. Convergence of simulations and analysis**			
1a. Is an evaluation presented in the text to show that the property being measured has equilibrated in the simulations (*e.g.* time-course analysis)?	☒	☐	Figure 3D and the subsection "*System construction and molecular dynamics simulations*"
1b. Then, is it described in the text how simulations are split into equilibration and production runs and how much data were analyzed from production runs?	☒	☐	In the subsection "*System construction and molecular dynamics simulations*"
1c. Are there at least 3 simulations per simulation condition with statistical analysis?	☐	☒	We performed a histogram analysis of water number distribution in the cavity using snapshots from a long trajectory. Statistical information is included in the analysis for an equilibrated system.
1d. Is evidence provided in the text that the simulation results presented are independent of initial configuration?	☒	☐	In the subsection "*System construction and molecular dynamics simulations*"
**2. Connection to experiments**			
2a. Are calculations provided that can connect to experiments (*e.g.* loss or gain in function from mutagenesis, binding assays, NMR chemical shifts, J-couplings, SAXS curves, interaction distances or FRET distances, structure factors, diffusion coefficients, bulk modulus and other mechanical properties, *etc.*)?	☒	☐	Figure 3D and the subsection "*The Resting VSD Structure Reveals a Deep Closed State of the Pore*" of the main text
**3. Method choice**			
3a. Do simulations contain membranes, membrane proteins, intrinsically disordered proteins, glycans, nucleic acids, polymers, or cryptic ligand binding?	☒	☐	Response not needed if **No**
3b. Is it described in the text whether the accuracy of the chosen model(s) is sufficient to address the question(s) under investigation (e.g. all-atom vs. coarse-grained models, fixed charge vs. polarizable force fields, implicit vs. explicit solvent or membrane, force field and water model, etc.)?	☒	☐	In the subsection "*System construction and molecular dynamics simulations*"
3c. Is the timescale of the event(s) under investigation beyond the brute-force MD simulation timescale in this study that enhanced sampling methods are needed?	☐	☒	
If **YES**, are the parameters and convergence criteria for the enhanced sampling method clearly stated?	☐	☐	
If **NO,** is the evidence provided in the text?	☒	☐	Figure 3D and the subsection "*System construction and molecular dynamics simulations*"

4. Code and reproducibility			
4a. Is a table provided describing the system setup that includes simulation box dimensions, total number of atoms, total number of water molecules, salt concentration, lipid composition (number of molecules and type)?	☒	☐	Supplementary Table 2
4b. Is it described in the text what simulation and analysis software and which versions are used?	☒	☐	In the subsection "*System construction and molecular dynamics simulations*"
4c. Are other parameters for the system setup described in the text, such as protonation state, type of structural restraints if applied, nonbonded cutoff, thermostat and barostat, etc.?	☒	☐	In the subsection "*System construction and molecular dynamics simulations*"
4d. Are initial coordinate and simulation input files and a coordinate file of the final output provided as supplementary files or in a public repository?	☒	☐	Provided as supplementary files
4e. Is there custom code or custom force field parameters?	☐	☒	Response not needed if **No**
If **YES**, are they provided as supplementary files or in a public repository?	☐	☐	
