# Supplementary material for: Structural basis of voltage-dependent gating in BK channels
Source: Nat Commun. 2025 Jul 1;16:5846. doi: 10.1038/s41467-025-60639-y (PMC12218146; doi:10.1038/s41467-025-60639-y)
Supplement: Supplementary file 2 — Description of Additional Supplementary Files [file 41467_2025_60639_MOESM2_ESM.pdf]

Description of Additional Supplementary Files

File Name: Supplementary Movie 1

Description: VSD upward change in the R2 rotamer orientation of approximately 6 Å while R3 reorients ~5 Å

File Name: Supplementary Movie 2

Description: Reorientation of D175 at S3 and D142 at S2, with state-dependent interactions between R2 and D175 and D142.

File Name: Supplementary Movie 3

Description: At 0 Ca^{2+}, residue Y212 (S4) is packed between the backbone nitrogen of I209 and R100 (S1) between S4 and S1

File Name: Supplementary Movie 4

Description: side-chain rotations of residues F304 and I308

File Name: Supplementary Data 1

Description:

MD trajectories

-initial and final coordinates

HOLE program analysis

-output
