# Supplementary material for: Structural basis of voltage-dependent gating in BK channels
Source: Nat Commun. 2025 Jul 1;16:5846. doi: 10.1038/s41467-025-60639-y (PMC12218146; doi:10.1038/s41467-025-60639-y)
Supplement: Supplementary file 8 — Reporting Summary [file 41467_2025_60639_MOESM8_ESM.pdf]

nature portfolio

Corresponding author(s): DAPR NCOMMS-24-20247A

Last updated by author(s): Apr 20, 2025

Reporting Summary

Nature Portfolio wishes to improve the reproducibility of the work that we publish. This form provides structure for consistency and transparency in reporting. For further information on Nature Portfolio policies, see our Editorial Policies and the Editorial Policy Checklist.

Statistics

For all statistical analyses, confirm that the following items are present in the figure legend, table legend, main text, or Methods section.

n/a	Confirmed	
☐	☒	The exact sample size (*n*) for each experimental group/condition, given as a discrete number and unit of measurement
☐	☒	A statement on whether measurements were taken from distinct samples or whether the same sample was measured repeatedly
☐	☒	The statistical test(s) used AND whether they are one- or two-sided<br>*Only common tests should be described solely by name; describe more complex techniques in the Methods section.*
☒	☐	A description of all covariates tested
☒	☐	A description of any assumptions or corrections, such as tests of normality and adjustment for multiple comparisons
☐	☒	A full description of the statistical parameters including central tendency (e.g. means) or other basic estimates (e.g. regression coefficient) AND variation (e.g. standard deviation) or associated estimates of uncertainty (e.g. confidence intervals)
☒	☐	For null hypothesis testing, the test statistic (e.g. *F*, *t*, *r*) with confidence intervals, effect sizes, degrees of freedom and *P* value noted<br>*Give P values as exact values whenever suitable.*
☒	☐	For Bayesian analysis, information on the choice of priors and Markov chain Monte Carlo settings
☒	☐	For hierarchical and complex designs, identification of the appropriate level for tests and full reporting of outcomes
☒	☐	Estimates of effect sizes (e.g. Cohen's *d*, Pearson's *r*), indicating how they were calculated

Our web collection on statistics for biologists contains articles on many of the points above.

Software and code

Policy information about availability of computer code

Data collection	For cryo-EM, automated data collection was performed using EPU (version 2.13.0.1546, Thermo Fisher). For electrophysiological experiments, data acquisition was carried out using pClamp (version 10.5.1, Molecular Devices).
Data analysis	Cryo-EM Data AnalysisRELION (v3.1.1) – Used for overall cryo-EM analysis, including particle polishing and classification.MotionCor2 (v1.4.0) – Applied for beam-induced motion correction of raw micrographs.CTFFIND4.1 (v4.1.5–4.1.9) – Used for contrast transfer function (CTF) estimation.SPHIRE-crYOLO (v1.7.0–1.8.0) – Automated particle picking.ResMap (v1.1.4) – Local resolution estimation of 3D density maps.COOT (v0.9.6) – Manual model building and adjustment.ISOLDE (v1.1–1.8) – Interactive flexible fitting of atomic models into density.Phenix (v1.14-3260) – Real-space refinement of atomic models.MolProbity (v4.5) – Structural validation and geometry assessment.HOLE (v2.0) – Calculation of pore radius profiles.UCSF ChimeraX (v1.3–1.9) – Visualization and preparation of structural figures.Molecular Dynamics SimulationsCHARMM-GUI (v3.8) – System construction and membrane embedding for simulations.VMD (v1.9.3) – Visualization and analysis of MD trajectories.NAMD (v3.0 alpha9) – Molecular dynamics simulations.Electrophysiology AnalysispCLAMP (Clampfit) (v10.5.1, Molecular Devices) – Data acquisition and analysis of patch-clamp recordings.Python (v3.10) – Used for plotting and statistical analysis of electrophysiological data.

For manuscripts utilizing custom algorithms or software that are central to the research but not yet described in published literature, software must be made available to editors and reviewers. We strongly encourage code deposition in a community repository (e.g. GitHub). See the Nature Portfolio guidelines for submitting code & software for further information.

Data

Policy information about availability of data

All manuscripts must include a data availability statement. This statement should provide the following information, where applicable:

- Accession codes, unique identifiers, or web links for publicly available datasets
- A description of any restrictions on data availability
- For clinical datasets or third party data, please ensure that the statement adheres to our policy

Cryo-EM density maps of aBK mutants have been deposited in the Electron Microscopy Data Bank under accession codes (EMDB):
EMD-46903 [https://www.ebi.ac.uk/pdbe/entry/emdb/EMD-46903] (R1Q-apo),
EMD-46901 [https://www.ebi.ac.uk/pdbe/entry/emdb/EMD-46901] (R1Q-Ca2+-bound),
EMD-46939 [https://www.ebi.ac.uk/pdbe/entry/emdb/EMD-46939] (R2Q-apo),
EMD-46918 [https://www.ebi.ac.uk/pdbe/entry/emdb/EMD-46918] (R2Q-Ca2+-bound),
EMD-46963 [https://www.ebi.ac.uk/pdbe/entry/emdb/EMD-46963] (R3Q-apo),
EMD-46956 [https://www.ebi.ac.uk/pdbe/entry/emdb/EMD-46956] (R3Q-Ca2+-bound), and
EMD-46961 [https://www.ebi.ac.uk/pdbe/entry/emdb/EMD-46961] (F304A-Ca2+-bound).
The atomic model of the aBK mutants has been deposited in the Protein Data Bank under accession code:
9DIC [https://doi.org/10.2210/pdb9DIC/pdb] (R1Q-apo),
9DI8 [https://doi.org/10.2210/pdb9DI8/pdb] (R1Q-Ca2+-bound),
9DJV [https://doi.org/10.2210/pdb9DJV/pdb] (R2Q-apo),
9DIT [https://doi.org/10.2210/pdb9DIT/pdb] (R2Q-Ca2+-bound),
9DKN [https://doi.org/10.2210/pdb9DKN/pdb] (R3Q-apo),
9DKF [https://doi.org/10.2210/pdb9DKF/pdb] (R3Q-Ca2+-bound), and
9DKL [https://doi.org/10.2210/pdb9DKL/pdb] (F304A-Ca2+-bound).

Previously published PDB used in this article for comparation purpose:
5TJI [http://doi.org/10.2210/pdb5TJI/pdb] (WT-apo),
5TJ6 [http://doi.org/10.2210/pdb5TJ6/pdb] (WT-Ca2+-bound),
6ND0 [http://doi.org/10.2210/pdb6ND0/pdb] (hBK-apo-mem1),
8GH9 [http://doi.org/10.2210/pdb8GH9/pdb] (hBK-apo-mem2),
6V5A [http://doi.org/10.2210/pdb6V5A/pdb] (hBK–L380P),
6V38 [http://doi.org/10.2210/pdb6V5A/pdb] (hBK-Ca2+-bound), and
6V3G [http://doi.org/10.2210/pdb6V3G/pdb] (hBK-apo).
The initial and final configurations of the molecular dynamics trajectories are provided in Supplementary Data File 1.
The source data underlying Figures 1b, and Figure 2b are provided in Supplementary Data File 2.

Research involving human participants, their data, or biological material

Policy information about studies with human participants or human data. See also policy information about sex, gender (identity/presentation), and sexual orientation and race, ethnicity and racism.

Reporting on sex and gender	*Use the terms sex (biological attribute) and gender (shaped by social and cultural circumstances) carefully in order to avoid confusing both terms. Indicate if findings apply to only one sex or gender; describe whether sex and gender were considered in study design; whether sex and/or gender was determined based on self-reporting or assigned and methods used. Provide in the source data disaggregated sex and gender data, where this information has been collected, and if consent has been obtained for sharing of individual-level data; provide overall numbers in this Reporting Summary. Please state if this information has not been collected. Report sex- and gender-based analyses where performed, justify reasons for lack of sex- and gender-based analysis.*
Reporting on race, ethnicity, or other socially relevant groupings	*Please specify the socially constructed or socially relevant categorization variable(s) used in your manuscript and explain why they were used. Please note that such variables should not be used as proxies for other socially constructed/relevant variables (for example, race or ethnicity should not be used as a proxy for socioeconomic status). Provide clear definitions of the relevant terms used, how they were provided (by the participants/respondents, the researchers, or third parties), and the method(s) used to classify people into the different categories (e.g. self-report, census or administrative data, social media data, etc.) Please provide details about how you controlled for confounding variables in your analyses.*
Population characteristics	*Describe the covariate-relevant population characteristics of the human research participants (e.g. age, genotypic information, past and current diagnosis and treatment categories). If you filled out the behavioural & social sciences study design questions and have nothing to add here, write "See above."*
Recruitment	*Describe how participants were recruited. Outline any potential self-selection bias or other biases that may be present and how these are likely to impact results.*
Ethics oversight	*Identify the organization(s) that approved the study protocol.*

Note that full information on the approval of the study protocol must also be provided in the manuscript.

Field-specific reporting

Please select the one below that is the best fit for your research. If you are not sure, read the appropriate sections before making your selection.

- [x] Life sciences
- [ ] Behavioural & social sciences
- [ ] Ecological, evolutionary & environmental sciences

For a reference copy of the document with all sections, see nature.com/documents/nr-reporting-summary-flat.pdf

Life sciences study design

All studies must disclose on these points even when the disclosure is negative.

Sample size	No statistical methods were used to predetermine sample sizes. For cryo-EM, particle numbers were determined empirically based on achieving high-resolution reconstructions, consistent with established practices in the field. For electrophysiological experiments, sample sizes were chosen based on prior studies and standard practice to ensure reproducibility and statistical significance (n-values are indicated in figure legends). Molecular dynamics simulations used representative structures with starting and ending configurations provided to illustrate relevant conformational states.
Data exclusions	No data were excluded from the analyses. All cryo-EM datasets, electrophysiological recordings, and molecular dynamics simulations that met quality and reproducibility criteria were included. Particles or trajectories failing standard quality control (e.g., poor CTF fit, low-resolution 2D classes, or unstable MD configurations) were removed based on predefined thresholds during data processing.
Replication	All key findings were confirmed across multiple independent experiments. Electrophysiological recordings were performed in at least three independent biological replicates, with consistent results. Cryo-EM structures were obtained from multiple datasets and reconstructions yielded reproducible conformations. Molecular dynamics simulations were repeated to ensure stability and convergence of structural features.
Randomization	Randomization was not applicable to this study, as no experimental groups were assigned. Sample selection for electrophysiological recordings, cryo-EM data collection, and molecular dynamics simulations was based on predefined quality criteria and standard experimental protocols.
Blinding	Blinding was not applicable to this study. Data collection and analysis for cryo-EM, electrophysiology, and molecular dynamics simulations were performed using automated or objective computational tools, where experimental outcomes were not subject to observer bias.

Reporting for specific materials, systems and methods

We require information from authors about some types of materials, experimental systems and methods used in many studies. Here, indicate whether each material, system or method listed is relevant to your study. If you are not sure if a list item applies to your research, read the appropriate section before selecting a response.

Materials & experimental systems

n/a	Involved in the study	
x		Antibodies
	x	Eukaryotic cell lines
x		Palaeontology and archaeology
x		Animals and other organisms
x		Clinical data
x		Dual use research of concern
x		Plants

Methods

n/a	Involved in the study	
x		ChIP-seq
x		Flow cytometry
x		MRI-based neuroimaging

Eukaryotic cell lines

Policy information about cell lines and Sex and Gender in Research

Cell line source(s)	sf9 cells from Thermo Fisher, catalog #12659017.<br>Adult female Xenopus laevis older than one year was used as the oocyte donor.
Authentication	None of the cell lines used in this study were authenticated.
Mycoplasma contamination	The cell lines were not tested for mycoplasma contamination.
Commonly misidentified lines (See ICLAC register)	None of the cell lines used in this study are listed as commonly misidentified.

Plants

Seed stocks	*Report on the source of all seed stocks or other plant material used. If applicable, state the seed stock centre and catalogue number. If plant specimens were collected from the field, describe the collection location, date and sampling procedures.*
Novel plant genotypes	*Describe the methods by which all novel plant genotypes were produced. This includes those generated by transgenic approaches, gene editing, chemical/radiation-based mutagenesis and hybridization. For transgenic lines, describe the transformation method, the number of independent lines analyzed and the generation upon which experiments were performed. For gene-edited lines, describe the editor used, the endogenous sequence targeted for editing, the targeting guide RNA sequence (if applicable) and how the editor was applied.*
Authentication	*Describe any authentication procedures for each seed stock used or novel genotype generated. Describe any experiments used to assess the effect of a mutation and, where applicable, how potential secondary effects (e.g. second site T-DNA insertions, mosiacism, off-target gene editing) were examined.*
